# Synthesis and Application of Optical Nanomaterials

**DOI:** 10.3390/nano14231904

**Published:** 2024-11-27

**Authors:** Bong-Hyun Jun

**Affiliations:** Department of Bioscience and Biotechnology, Konkuk University, Seoul 143-701, Republic of Korea; bjun@konkuk.ac.kr

The field of optical nanomaterials stands at the forefront of technological innovation, driving advancements across a spectrum of scientific disciplines and industries [1,2,3]. These materials are pivotal due to their unique ability to manipulate light at the nanoscale, facilitating breakthroughs in areas ranging from photonic devices and sensors to energy harvesting and biomedical applications [4,5,6]. The synthesis and tailored application of these nanomaterials not only enhance the performance and efficiency of existing technologies but also open the door to novel applications that were previously unattainable. As such, the research collected in this Special Issue, “Synthesis and Application of Optical Nanomaterials”, encapsulates a comprehensive exploration of cutting-edge methodologies, characterizations, and implementations, showcasing the profound impact and vast potential of these materials in shaping the future of technology and innovation.

Launching this inaugural edition, we present thirteen outstanding papers that provide original contributions detailing the synthesis, modification, and innovative applications of optical nanomaterials. Due to the critical importance of these materials in advancing technology and science, each paper offers a glimpse into the most recent advances, highlighting the dynamic and rapidly evolving nature of this field. This collection reflects a diverse array of pioneering research, establishing the role of optical nanomaterials as essential components of modern scientific inquiry and technological development.

The unique and significant properties of plasmonic materials are pivotal; this is exemplified in the research discussed in our featured papers, which delve into their innovative applications and transformative potential. These metal-embedded silica NPs significantly boost applications like plasmon resonance and Raman scattering, with potential in fields such as biology and medicine [7,8]. Cho et al. review silica nanoparticles (NPs) as biocompatible templates for embedding noble metals, enhancing optical properties, and overcoming limitations of metal NPs alone [9]. Hahm et al. discuss optically active NPs embedded in silica, modified with organic ligands to maintain their optical properties and stability. The study introduces a versatile silica encapsulation method using TMPS for non-hydrophilic NPs like quantum dots, ensuring their optical performance without significant changes [10]. Kim et al. introduce a novel SALDI-MS method using silica@gold core–shell hybrid materials with nanogap-rich shells to enhance the detection of small molecules [11]. By optimizing the gold shell thickness, this method offers improved detection limits, reproducibility, and salt tolerance, making it ideal for small molecule analysis. Ali et al. explore plasmonic nanostructures as narrowband perfect absorbers, achieving up to 99.94% light absorption in the near-infrared spectrum. The tunability of these structures, through adjustments in geometry and dielectric layers, makes them highly sensitive and ideal for biosensing and environmental detection [12]. Li et al. investigate how surface plasmon resonance (SPR) can enhance photodetectors by using surface roughness and aluminum nanoparticles on ZnO films [13]. The study shows that increasing surface roughness significantly boosts the photodetector’s response, enhancing SPR and improving responsivity by three orders of magnitude. Zhang et al. review the applications of plasma nanoparticles in fields like biosensing and optical imaging, focusing on local surface plasmon resonance (LSPR) [14]. They discuss how modifying the dielectric environment, electromagnetic coupling, and charge transfer enhance LSPR performance in plasmonic nanoprobes, advancing their use in detection and analysis.

This collection introduces a wide range of materials and methods, highlighting the synthesis, modification, and application of nanostructured materials such as metal oxides, polymers, phosphors, and organic crystals, each with unique optical, mechanical, and electronic properties. Through techniques like solvothermal synthesis, thermal annealing, and laser ablation these studies show how nanomaterials can be tailored for advanced applications in chemical sensing, biosensing, material removal, and visual mechanical sensing, demonstrating their potential across various technological fields. Maciulis et al. review the advantages of nanostructured metal oxides, including large surface areas and tunable properties, making them essential for chemical sensors and biosensors [15]. They classify these nanostructures by their dimensions and provide a detailed analysis of synthesis methods and future sensor integration. Ouyang et al. synthesized zirconia (ZrO_2_) nanoparticles using varying conditions, revealing how synthesis affects crystal structures and grain sizes [16]. The study highlights the role of oxygen vacancies in stabilizing phases and influencing optical properties, expanding the potential applications of ZrO_2_. Chauhan et al. improve qPCR performance by integrating PEG-nGO, which enhances efficiency and specificity by reducing nonspecific amplification [17]. Their PENGO-qPCR method demonstrates significantly higher sensitivity for detecting viral RNA compared to traditional qPCR setups. Gamaly et al. explore material removal using ultra-short laser pulses, minimizing energy loss and enabling high-precision nanomachining [18]. The study compares this mechanism to the photo effect and nanocluster Coulomb explosions and investigates experimental approaches for detecting ablation via THz radiation. Shchur et al. synthesized nanosized benzil crystals embedded in SiO_2_ matrices, showing that their structural and vibrational properties remain stable despite space confinement. Their ab initio calculations confirmed the mechanical and dynamical stability of the benzil lattice [19]. Dabert et al. investigated the effects of thermal annealing on silver NP@polymer coatings [20]. The reorganization of nanoparticles during annealing enhances electrical conductivity, hydrophobicity, and reflectivity, with promising applications in energy-efficient reflectors and microdevices. Wang et al. synthesized CaZnOS^+^, Sm^3+^ phosphor, which exhibits tunable multicolor mechanoluminescence under mechanical stimulation [21]. This material, mixed with PDMS for practical applications like handwriting identification, offers excellent performance and color tunability for visual mechanical sensing.

In conclusion, the research presented in this collection underscores the immense potential of optical nanomaterials to revolutionize a wide range of industries and scientific domains. Each paper provides valuable insights into the synthesis, characterization, and application of these materials, showcasing their versatility and transformative potential. As the field continues to evolve, the innovative methodologies and applications highlighted in this issue will undoubtedly pave the way for future technological breakthroughs and a deeper understanding of the nanoscale world.
